# Tomorrow's Health Data Scientists in the Making: 2 Years of Training & Capacity Building Activities with the Farr Institute

**DOI:** 10.23889/ijpds.v1i1.299

**Published:** 2017-04-19

**Authors:** Athanasios Anastasiou, Georgina Moulton, Colin McCowan, Paul Taylor, Catharine Goddard

**Affiliations:** Swansea University Medical School; University of Manchester; University of Glasgow; University College London; University of Dundee

## Objectives

The poster will showcase the Training & Capacity Building Programme (TCBP) established by the Farr network that is available to PhD students and Early Career Researchers (ECRs) both from within and outside the network.

## Approach

The authors are using a mixed-methods approach to present the current state of the art that has motivated the structure of the TCBP and how the programme has been received by students, academic and industry leaders.

Since 2014, the Farr network education leads have worked on a unique education programme that aims to equip students with key professional and methodological skills required by the roles they are likely to take in their future careers.

The content of the programme has been informed by current research in the fields of data linkage and analysis of big datasets as well as the immediate experience of researchers, and healthcare and industry leads working actively in the field.

We have focussed on developing a community of practice through the provision of an environment that has enabled students to share experiences and knowledge and to start building their own networks of collaboration across the Farr centres.

## Results

The Farr network of PhD students comprises of approximately 66 students from a variety of backgrounds including bioinformatics, computer science, epidemiology, mathematics, psychology and statistics.

The Farr network hosted 2 main events in 2015 that provided the foundation for 34 PhD students to come together to develop skills in written and oral communication, Public and Patient Involvement (PPI), problem-solving, requirements gathering, data visualisation and spatial data analysis.

Formal feedback collected from each event suggests that the programme is received well by students who see it as a set of activities that complement their specialised PhD related education. Feedback from supervisors and future employers also suggests that this programme has facilitated the development of skills that are useful for their PhD and employment.

## Conclusion

The Farr network has laid the foundation for the community of practice around the analysis of real-world health datasets across the UK, and will continue to cultivate this through the enrichment of the programme. 

